# A Novel Zero Velocity Interval Detection Algorithm for Self-Contained Pedestrian Navigation System with Inertial Sensors

**DOI:** 10.3390/s16101578

**Published:** 2016-09-24

**Authors:** Xiaochun Tian, Jiabin Chen, Yongqiang Han, Jianyu Shang, Nan Li

**Affiliations:** School of Automation, Beijing Institute of Technology, Beijing 100081, China; 3120130358@bit.edu.cn (X.T.); chenjiabin@bit.edu.cn (J.C.); 3120130357@bit.edu.cn (J.S.); 3120130376@bit.edu.cn (N.L.)

**Keywords:** pedestrian navigation system (PNS), adaptive ZVI detection, SPWVD, gait frequency, ZUPT

## Abstract

Zero velocity update (ZUPT) plays an important role in pedestrian navigation algorithms with the premise that the zero velocity interval (ZVI) should be detected accurately and effectively. A novel adaptive ZVI detection algorithm based on a smoothed pseudo Wigner–Ville distribution to remove multiple frequencies intelligently (SPWVD-RMFI) is proposed in this paper. The novel algorithm adopts the SPWVD-RMFI method to extract the pedestrian gait frequency and to calculate the optimal ZVI detection threshold in real time by establishing the function relationships between the thresholds and the gait frequency; then, the adaptive adjustment of thresholds with gait frequency is realized and improves the ZVI detection precision. To put it into practice, a ZVI detection experiment is carried out; the result shows that compared with the traditional fixed threshold ZVI detection method, the adaptive ZVI detection algorithm can effectively reduce the false and missed detection rate of ZVI; this indicates that the novel algorithm has high detection precision and good robustness. Furthermore, pedestrian trajectory positioning experiments at different walking speeds are carried out to evaluate the influence of the novel algorithm on positioning precision. The results show that the ZVI detected by the adaptive ZVI detection algorithm for pedestrian trajectory calculation can achieve better performance.

## 1. Introduction

Pedestrian navigation, having received more and more attention from researchers in recent years, is an important branch in the field of navigation. PNS can help personnel in missions to identify their positions in real time and to get in touch with the command center; thus the safety of the emergency rescue personnel can be greatly guaranteed in an unknown environment. In addition, in places such as an airport, theatre, underground parking, other large public places and modern cities with tall buildings, it is necessary for pedestrians to identify their positions and find targets.

At present, technologies suitable for pedestrian navigation can be divided into positioning technology based on the Global Navigation Satellite System (GNSS), positioning technology based on the radio frequency (RF) signal, as well as positioning technology based on self-contained sensors. The first kind is relatively mature, but the positioning accuracy is affected in indoor environments or outdoor environments with tall buildings and trees. The PNS based on the RF signal (such as RFID [1], UWB [2], etc.) requires pre-installing signal transmitting equipment in the positioning area, which is costly and has limited application range. Compared with the other two positioning technologies, PNS based on self-contained sensors has the advantages of strong autonomy and independence because it mainly adopts sensors, such as accelerometers, gyroscopes and magnetometers, to calculate pedestrian position information. Considering the limited load-carrying ability of pedestrians, in this paper, self-contained sensors are adopted to design PNS by installing them on the pedestrian’s foot, and the pedestrian’s position is obtained by integrating the inertial sensors output. However, low-cost inertial sensors have low performance, and the acceleration error produces a position error that grows cubically over time [3]. Therefore, it is very important to eliminate the error accumulation of PNS for the improvement of positioning accuracy.

During pedestrian’s walking process, there is a period (about 0.3–0.4 s [4]) when the sole fully contacts with ground and the foot velocity approximates zero. This period is usually called ZVI. According to the periodic existence of ZVI in pedestrian gait, ZUPT can be adopted for clearing the position error periodically, where the core of ZUPT is accurately detecting the ZVI in the pedestrian gait. Many scholars have carried out research on ZVI detection methods; for example Zhou et al. [5] used a shoe-mounted radar to detect the ZVI; Bebek et al. [6] measured the ZVI by adopting a high-resolution pressure sensor for assistance; Zhou et al. [7] detected the ZVI by embedding an RF sensor in the pedestrian’s shoe. Generally speaking, the above-mentioned methods can better detect the ZVI, but they all need additional sensors as assistance to realize the ZVI detection, adding the cost of a PNS. In addition, the PNS based on self-contained sensors can only use the output of accelerometers and gyroscopes to detect the ZVI without the assistance of external sensors. Commonly-used ZVI detection methods for PNS with self-contained sensors include the acceleration magnitude method [8], the angular velocity magnitude method [9,10,11,12], the moving variance method [13], a combination of the above methods [14,15,16], and so on; all of these methods have a common characteristic that the ZVI is detected by setting a threshold.

The thresholds in the traditional ZVI detection methods take on fixed values. When a pedestrian walks at a constant gait frequency, the ZVI can be accurately detected by setting a fixed threshold; however, actually, pedestrians move randomly and cannot maintain a constant gait frequency all of the time. Previous research work has found out that different gait frequencies corresponds to different optimal thresholds. Therefore, when a pedestrian walks at a changing gait frequency, the adoption of a fixed threshold leads to missed or false detection in the ZVI detection results. In order to solve this problem, a novel adaptive ZVI detection algorithm based on SPWVD-RMFI is proposed in this paper, and the function relationships between the optimal ZVI detection thresholds and the gait frequency are established experimentally. During the walking process, the gait frequency is extracted in real time by adopting SPWVD-RMFI, and in the meantime, by using the function relationships between the thresholds and the gait frequency, the optimal thresholds corresponding to the current gait frequency can be obtained; thus, the adaptive adjustment of thresholds with gait frequency is realized, and the purpose of accurately detecting ZVI is achieved.

The remainder of this paper is organized as follows: the architecture of the pedestrian navigation algorithm is described in Section 2; the characteristics of pedestrian gait are analyzed in Section 3, and an adaptive ZVI detection algorithm based on SPWVD-RMFI is designed, as well; the contrast experiments of ZVI detection, as well as pedestrian trajectory positioning experiments are carried out in Section 4. In the meantime, the performance of the proposed algorithm in this paper is assessed; in the last section the research results provided by this paper are summarized.

## 2. Pedestrian Navigation Algorithm Architecture

**Figure 1:**
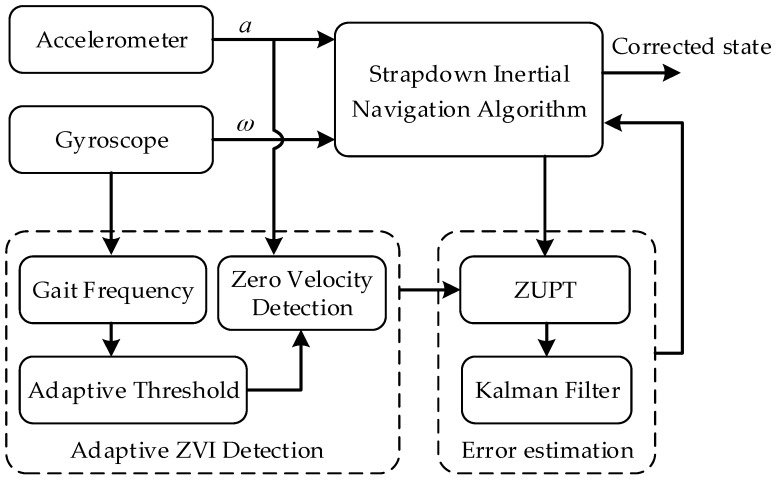
Pedestrian navigation algorithm architecture.

In this paper, the PNS algorithm based on self-contained sensors contains the strapdown inertial navigation algorithm module, the adaptive ZVI detection module and the error estimation module. The algorithm architecture is shown in Figure 1.

### 2.1. Strapdown Inertial Navigation Algorithm Module

The strapdown inertial navigation algorithm of PNS is similar to the traditional strapdown inertial navigation algorithm. The navigation calculation process mainly includes attitude update, velocity update and position update. The navigation coordinate system (*n*-frame) is defined as north-east-down, which is consistent with the local geographic coordinate system. In general, the motion range of the pedestrian is relatively small, so the real-time position and trajectory will be shown in the tangential frame in this paper. After collecting the IMU output data, the angular rate can be used to update the attitude matrix and calculate the attitude angle. On this basis, transforming the specific force measured in the body coordinate system (*b*-frame) to the navigation coordinate frame and removing the harmful acceleration, then the speed information can be obtained by integrating the acceleration once, and the position information can be obtained by integrating the acceleration again. The basic equations of the strapdown inertial navigation system (SINS) can be expressed as follows:

(1) $$\begin{array}{l} {\dot{C}}_{b}^{n}\;=\;C_{b}^{n}\left(\omega_{nb}^{b}\times \right)\\ {\dot{V}}^{n}\;=\;C_{b}^{n}f^{b}\;-\;\left(2\omega_{ie}^{n}+\omega_{en}^{n}\right)\;\times \;V^{n}\;+\;g^{n}\\ {\dot{P}}^{n}\;=\;V^{n} \end{array}$$

where n represents the *n*-frame and b stands for the *b*-frame, $C_{b}^{n}$ is the attitude transformation matrix from the *b*-frame to the *n*-frame, V is the velocity and P is the position. $\omega_{nb}^{b}$ represents the turn rate of the *b*-frame with respect to the *n*-frame; $f^{b}$ is the specific force; $\omega_{ie}^{n}$ is the Earth rotation angular rate; $\omega_{en}^{n}$ is the rotation angular rate of the *n*-frame with respect to the Earth frame; and $g^{n}$ is the Earth gravitational field vector, which approximates a constant within a range on Earth.

### 2.2. Adaptive ZVI Detection Module

The adaptive ZVI detection module is used to detect the zero velocity information in the pedestrian gait and to provide a trigger condition for the Kalman filter. According to the characteristics that the pedestrian’s walking speed changes over time, an adaptive ZVI detection algorithm based on SPWVD-RMFI is proposed in this paper. The novel algorithm firstly uses the SPWVD-RMFI method to extract the gait frequency by processing the gyroscope output, and then, the function relationships between the ZVI detection thresholds and the gait frequency are established to calculate the optimal ZVI detection thresholds in real time. On this basis, the ZVI at different walking speeds can be detected adaptively and accurately. Detailed research contents are described in Section 3.

### 2.3. Error Estimation Module

As low-cost inertial sensors have a large bias, the navigation parameters obtained by the SINS algorithm often contain error terms. The error estimation module uses the ZUPT to assist the Kalman filter to estimate the system state errors. The error state vectors are defined as follows:

(2) $$\delta x\;=\;\left[\begin{array}{ccccc} \delta \phi^{n} & \delta V^{n} & \delta P^{n} & \varepsilon^{b} & \nabla^{b} \end{array}\right]$$

where $\delta \phi^{n}$ is the attitude error vector, $\delta V^{n}$ is the velocity error vector, $\delta P^{n}$ is the position error vector, $\varepsilon^{b}$ is the gyroscope bias error vector and $\nabla^{b}$ is the accelerometer bias error vector.

The discrete system state equation is:

(3) $$\delta x_{k\;+\;1}\;=\;\Phi_{k}\delta x_{k}\;+\;w_{k}$$

where $\delta x_{k}$ is the system state at time k, $\Phi_{k}$ is the state transition matrix and $w_{k}$ is the process noise. In this study, the expression of the state transition matrix is:

(4) $$\Phi_{k}\;=\;\left[\begin{array}{ccccc} I_{3\;\times \;3} & 0_{3\;\times \;3} & 0_{3\;\times \;3} & \Delta t\cdot C_{b}^{n} & 0_{3\;\times \;3}\\ \Delta t\cdot f^{n}\times & I_{3\;\times \;3} & 0_{3\;\times \;3} & 0_{3\;\times \;3} & \Delta t\cdot C_{b}^{n}\\ 0_{3\;\times \;3} & \Delta t\cdot I_{3\;\times \;3} & I_{3\;\times \;3} & 0_{3\;\times \;3} & 0_{3\;\times \;3}\\ 0_{3\;\times \;3} & 0_{3\;\times \;3} & 0_{3\;\times \;3} & \left(1\;-\;\frac{\Delta t}{\tau_{g}}\right)I_{3\;\times \;3} & 0_{3\;\times \;3}\\ 0_{3\;\times \;3} & 0_{3\;\times \;3} & 0_{3\;\times \;3} & 0_{3\;\times \;3} & \left(1\;-\;\frac{\Delta t}{\tau_{a}}\right)I_{3\;\times \;3} \end{array}\right]$$

where $\tau_{g}$ and $\tau_{a}$ are the correlation times of the gyroscopes and accelerometers, respectively, Δt stands for the sample time interval and $f^{n}\times$ is the skew symmetric matrix constructed by the specific force in the *n*-frame.

When the ZVI is detected, the velocity error can be obtained by doing a subtraction operation between the velocity calculated by the SINS and the velocity in the ZVI; then feeding the velocity error into the Kalman filter for measurement update. The measurement equation of the system is:

(5) $$\delta z_{k}\;=\;H\delta x_{k}\;+\;v_{k}$$

where $\delta z_{k}$ is the error measurement at time k, H is the measurement matrix and $v_{k}$ is the measurement noise. For the ZUPT-based Kalman filter, the expression of the measurement matrix H is as follows:

(6) $$H\;=\;\left[\begin{array}{lllll} 0_{3\;\times \;3} & I_{3\;\times \;3} & 0_{3\;\times \;3} & 0_{3\;\times \;3} & 0_{3\;\times \;3} \end{array}\right]$$

When the measurement update is performed, the system state error is estimated at every detected zero velocity; then, the navigation parameters and the inertial data can be corrected by feeding the estimated error back to the strapdown inertial navigation algorithm module.

In practical applications, the filter stability can be improved by tuning the parameters in the Kalman filter, which mainly include the state estimation covariance matrix *P*, the process noise covariance matrix *Q* and the measurement noise covariance matrix *R*. In this study, *P* and *Q* both are diagonal 15 × 15 matrices with elements as $P=diag\;\left[\begin{array}{lllll} 0_{1\;\times \;3}, & 0_{1\;\times \;3}, & 0_{1\;\times \;3}, & (1\;\times \;10^{-2})\;I_{1\;\times \;3}, & (1\;\times \;10^{-2})\;I_{1\;\times \;3} \end{array}\right]$ and $Q=diag\;\left[\begin{array}{lllll} (1\;\times \;10^{-4})\;I_{1\;\times \;3}, & (1\;\times \;10^{-4})\;I_{1\;\times \;3}, & 0_{1\;\times \;3}, & 0_{1\;\times \;3}, & 0_{1\;\times \;3} \end{array}\right]$, respectively. *R* is a diagonal 3 × 3 matrix with elements as $R\;=\;{\left(0.02\text{ m/s}\right)}^{2}I_{3\;\times \;3}$. After tuning the Kalman filter parameters, stable experiment results can be obtained for the PNS.

## 3. Adaptive ZVI Detection Algorithm Based on SPWVD-RMFI

The PNS based on foot-mounted inertial sensors employs the ZUPT method to estimate and correct the system error, but the premise of ZUPT is to analyze the pedestrian’s gait characteristics effectively and to detect the ZVI correctly.

### 3.1. Gait Characteristics Analysis

**Figure 2:**
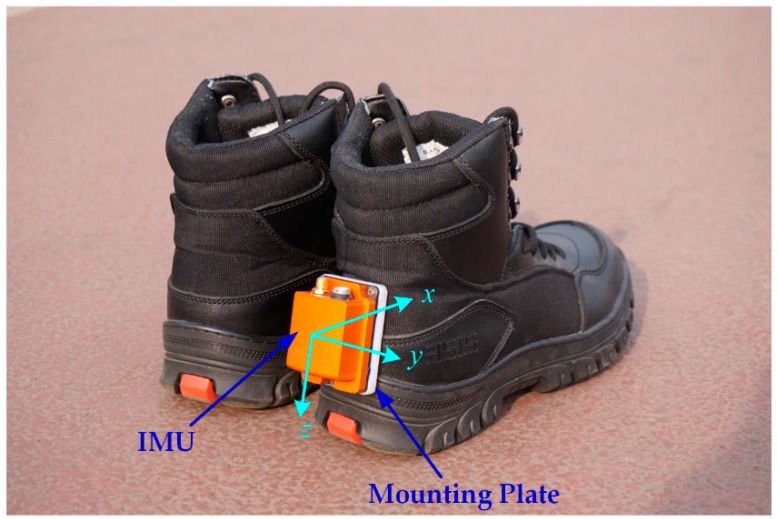
Pedestrian navigation shoe based on the IMU.

The pedestrian navigation shoe based on self-contained sensors is shown in Figure 2, where all of the sensors are integrated in a structure to constitute an inertial measurement unit (IMU). The IMU is fixed on a mounting plate on the heel part of the right shoe. The sensors in the x axis and the y axis measure the longitudinal direction and lateral direction motion parameters, respectively; the motion parameters in the vertical direction are measured by the sensors in the z axis.

**Figure 3:**
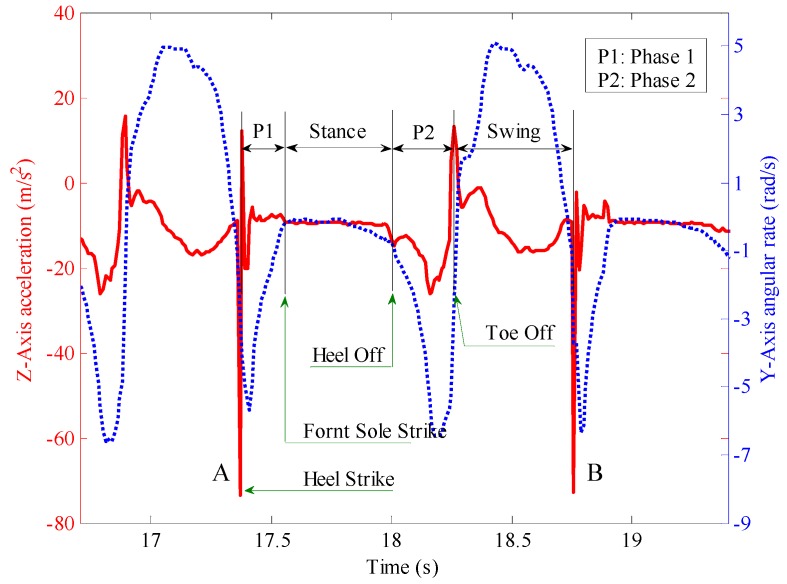
Pedestrian gait cycle.

A complete gait cycle shown in Figure 3 is obtained by using the navigation shoe to collect the inertial parameters of a pedestrian’s right foot during walking. The red solid line stands for the z axis accelerometer output, and the blue dotted line represents the y axis gyroscope output. The complete gait cycle between A and B in the figure is divided into four stages, which respectively are P1, stance, P2 and swing. P1 stands for the process from the heel striking the ground to the front sole striking the ground; during this period, the foot turns around the −y axis, and the gyroscope output is negative. Meanwhile, the output of accelerometer reaches the maximum when the heel strikes the ground. After the front sole contacts the ground completely, there is a period during which the sensors’ outputs are approximately constant (the gyroscope output is approximately zero, and the accelerometer output is approximately the gravitational acceleration). This period is also called ZVI (the stance phase shown in Figure 3). After the ZVI, the lift foot stage, P2, starts from the heel of the right foot lifting off the ground to the moment of toe off, during which the gyroscope output remains negative. After that, the right foot lifts off the ground, the leg begins to swing and the body moves forward (the swing phase shown in Figure 3). The gyroscope output corresponding to the gait change in the swing phase is positive. After the swing phase, the heel of the right foot strikes the ground again, which marks the beginning of another gait cycle.

### 3.2. Adaptive ZVI Detection

The analysis of the pedestrian’s gait characteristics in Section 3.1 indicates that the output of each sensor in ZVI has a significant difference from other motion stages of the gait cycle, namely that the gyroscope output approximates zero and that the acceleration output in the vertical direction is approximately the gravitational acceleration. According to these obvious features, the ZVI can be extracted by adopting the zero velocity detection method. The adaptive ZVI detection algorithm based on SPWVD-RMFI in this paper mainly consists of two parts: one part is the gait frequency analysis method based on SPWVD-RMFI, which can accurately extract the gait frequency during pedestrian’s walking process at any time; the other part is an adaptive ZVI detection algorithm, which can adaptively set the threshold for ZVI detection according to the change of pedestrian gait frequency and finally achieve the accurate detection of ZVI. Details are described as follows.

#### 3.2.1. Gait Frequency Extraction Based on SPWVD-RMFI

The analysis in Section 3.1 shows that during the pedestrian’s walking process, the gyroscope signal of the *y* axis can reflect the periodic change of the pedestrian gait completely and clearly. Therefore, the gait frequency at any time can be obtained by adopting a time-frequency analysis method. Commonly-used time-frequency analysis methods, such as short time Fourier transform (STFT) [17] and Wigner–Ville distribution (WVD) [18], can obtain the signal’s time-frequency information, but both methods have their own deficiencies: the STFT has a lower calculation precision; the WVD is unable to suppress the cross interference items [19]. The SPWVD adopted in this study is an improved WVD, which employs two independently controlled smooth window functions g(t) and h(τ) respectively in the time domain and frequency domain. The SPWVD is a time-frequency analysis method with higher performance in time-frequency focus; it can effectively suppress the cross interference items and improve the extraction precession of time-frequency information simultaneously. The SPWVD expression of continuous signal x(t) is:

(7) $$SPWVD(t,f)\;=\;\int_{-\infty }^{\infty }h(\tau )\int_{-\infty }^{\infty }g(\varepsilon \;-\;t)\cdot x(\varepsilon \;+\;\frac{\tau }{2})\cdot x^{*}(\varepsilon \;-\;\frac{\tau }{2})\;d\varepsilon e^{-j2\pi f\tau }d\tau$$

where,

(8) $$WVD(t,f)\;=\;\int_{-\infty }^{\infty }x(t\;+\;\frac{\tau }{2})\cdot x^{*}(t\;-\;\frac{\tau }{2})e^{-j2\pi f\tau }d\tau$$

where t is the time of x(t), f is the frequency of x(t) and $x^{*}(t)$ is the complex conjugate of x(t).

Because the sensor output is discrete rather than continuous, the continuous time signal x(t) needs to be discretized. Then, the discrete SPWVD can be expressed as:

(9) $$SPWVD(n,\hat{f})\;=\;\sum_{m=-(Q_{1}-1)/2}^{(Q_{1}-1)/2}h(m)e^{-j2\pi km/N}\sum_{p=-(Q_{2}-1)/2}^{(Q_{2}-1)/2}g(p)\cdot x(n\;-\;p\;+\;m)\cdot x^{*}(n\;-\;p\;-\;m)$$

where $Q_{1}$ and $Q_{2}$ are the window lengths of h(k) and g(n) respectively. n, $\hat{f}$, p and m are the discretization forms of the continuous variables t, f, ε and τ.

**Figure 4:**
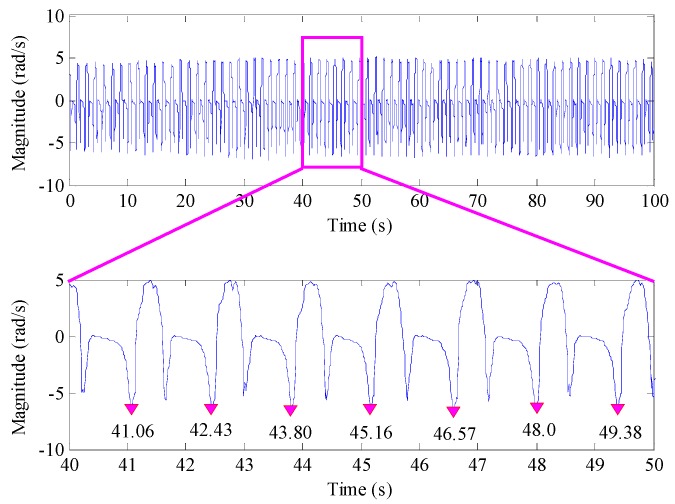
The time domain waveform of the *y* axis gyroscope output and its local enlarged drawing.

**Figure 5:**
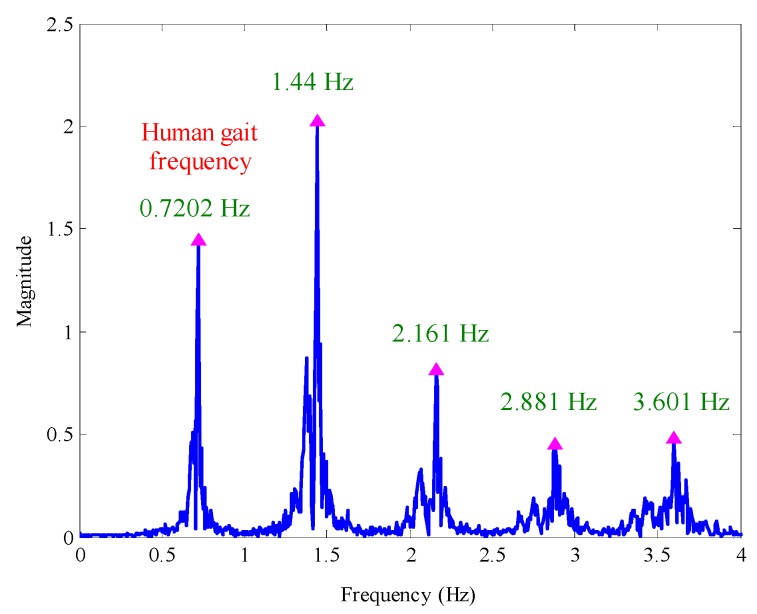
The Fourier spectrum of the *y* axis gyroscope output.

Collecting a group of *y* axis gyroscope output data during the walking process, the time domain waveform of the data is shown in Figure 4. It can be seen that the pedestrian’s walking speed is relatively stable, and the local enlarged drawing shows that the pedestrian gait frequency is about 0.72 Hz. After implementing Fourier transform, the amplitude-frequency characteristic of the *y* axis gyroscope output is shown in Figure 5, in which the frequency 1.44 Hz is the cyclic component with the strongest energy, and it is approximately to two-times the pedestrian gait frequency (0.7202 Hz). Meanwhile, other frequency components with strong energy are 2.161 Hz, 2.881 Hz and 3.601 Hz, which are approximately three-times, four-times and five-times the pedestrians gait frequency, respectively. This shows that multiple frequencies exist in the *y* axis gyroscope output.

**Figure 6:**
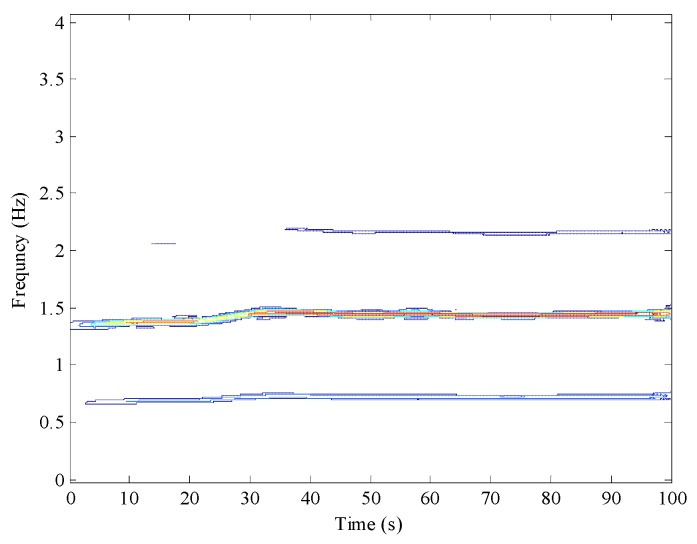
The SPWVD of the *y* axis gyroscope output.

Furthermore, the SPWVD was used to extract the time-frequency information of the *y* axis gyroscope output. Figure 6 is the time-frequency spectrum of the *y* axis gyroscope output. The color enclosing lines represent the signal amplitude at the corresponding time and frequency, and the same color enclosing lines represent the distribution of the frequency with the same signal amplitude. It is very clear that the time-frequency spectrum contains multi-peak spectral lines and that the maximum signal amplitude appears within the range of 1.3–1.55 Hz. Hence, if directly extracting the ridge line of the spectrum to obtain the time-frequency information, all of the multiple frequency constituents are interference items, which will make it difficult to identify the gait frequency. In addition, Figure 5 and Figure 6 show that the frequency doubling energy is higher than the gait frequency energy and that the frequency doubling is a range rather than a value, which makes the features of the gait frequency less prominent and increases the extraction difficulty.

**Figure 7:**
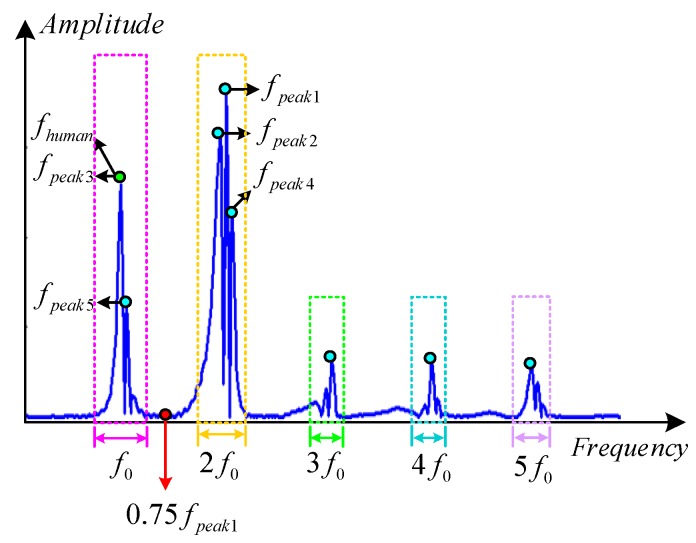
Principle diagram of the time-frequency extraction method based on SPWVD-RMFI.

In order to accurately identify the pedestrian gait frequency and effectively filter the multiple frequency constituents, this paper puts forward a time-frequency information extraction method based on SPWVD-RMFI, whose principle diagram is shown in Figure 7. The $f_{0}$, $2f_{0}$, $3f_{0}$, $4f_{0}$ and $5f_{0}$ are the ranges of the gait frequency, frequency doubling, frequency tripling, frequency quadrupling and frequency quintupling, respectively, of the *y* axis gyroscope output. Among those frequency ranges, the frequency doubling has the highest energy intensity, and the gait frequency has the second highest energy intensity. In addition, multi-peaks may appear in all of the multiple frequency ranges. Therefore, the pedestrian gait frequency $f_{human}$ could be the second or the N-th frequency with the highest energy intensity. The size of N depends on the number and the energy intensity of the multi-peaks in the frequency doubling range. However, all of the multiple frequencies and the number of the multi-peaks contained in the multiple frequency ranges are unknown during walking. Hence, under the premise of unknown gait frequency range $f_{0}$, how to extract the pedestrian gait frequency $f_{human}$ accurately and intelligently is a daunting task. Therefore, the time-frequency extraction method based on SPWVD-RMFI is proposed in this paper, and its principle is listed as follows:

(1) Using the SPWVD to extract the time-frequency spectral line of the *y* axis gyroscope output.
(2) Extract the frequency $f_{peak1}$ corresponding to the largest peak in the first time-frequency spectral line, as is shown in Figure 7; $f_{peak1}$ is in the range of frequency doubling ($2f_{0}$).
(3) Extract the frequency $f_{peak2}$ corresponding to the second largest peak in the first time-frequency spectral line, and judge the relationship between $f_{peak2}$ and $0.75f_{peak1}$. If $f_{peak2}\;<\;0.75f_{peak1}$, it indicates that $f_{peak2}$ is in the range of one time frequency, namely that $f_{peak2}$ is the gait frequency $f_{human\;1}$ corresponding to time $t_{1}$ during the walking process; otherwise, if $f_{peak2}\;>\;0.75f_{peak1}$, it indicates that $f_{peak2}$ is still in the frequency doubling range, which means that it is necessary to continue to extract the frequency $f_{peak\;N}$ corresponding to the N-th largest peak in the first time-frequency spectral line; meanwhile, the relationship between $f_{peak\;N}$ and $0.75f_{peak1}$ needs to be judged according to the above-mentioned steps until the judge condition $f_{peak\;N}\;<\;0.75f_{peak1}$ is fulfilled. At this moment, $f_{peak\;N}$ is the gait frequency $f_{human\;1}$ corresponding to time $t_{1}$ during the walking process. The N in $f_{peak\;N}$ is an integer greater than or equal to one.
(4) Analyze other time-frequency spectral lines in (1) according to Steps (2)–(3); then, the gait frequency $f_{human\;i}$ of any other time $t_{i}$ during the walking process can be obtained, where i is an integer greater than or equal to one.

**Figure 8:**
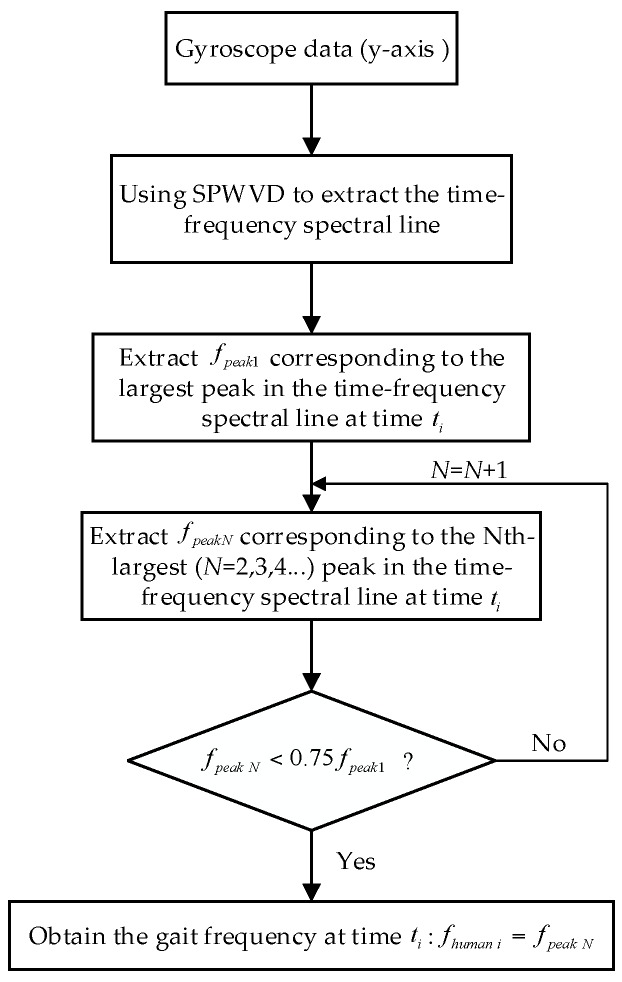
Flow chart of the time-frequency extraction method based on SPWVD-RMFI.

According to the above description, the principle of the time-frequency extraction method based on SPWVD-RMFI can be shown by the flow chart in Figure 8.

During the walking process, the SPWVD-RMFI method proposed in this paper can extract the gait frequency correctly, which provides the foundation for the thresholds’ selection in the adaptive ZVI detection algorithm.

#### 3.2.2. Adaptive ZVI Detection

On the basis of the gait frequency obtained by the SPWVD-RMFI method in Section 3.2.1, this section designs an adaptive ZVI detection algorithm. Through many experiments and statistical analysis, the thresholds at different walking speeds are obtained. Meanwhile, the function relationships between the optimal thresholds and the gait frequency are established. Furthermore, the algorithm can calculate the optimal ZVI detection thresholds based on the real-time gait frequency, and finally, the adaptive ZVI detection is achieved. The principle of the adaptive ZVI detection algorithm is as follows.

Define $\left|a(t_{i})\right|$ as the magnitude of the acceleration and $\sigma_{a}(t_{i})$ as the moving variance of the acceleration. During the walking process, they can be calculated by the following two equations:

(10) $$\left|a(t_{i})\right|=\sqrt{a_{x}{\left(t_{i}\right)}^{2}+a_{y}{\left(t_{i}\right)}^{2}+a_{z}{\left(t_{i}\right)}^{2}}$$

(11) $$\sigma_{a}(t_{i})=\sqrt{\frac{1}{n}{\sum_{i=m}^{m+w-1}\left(\left|a(t_{i})\right|-\bar{\left|a_{w}\right|}\right)}^{2}}$$

where $a_{x}(t_{i})$, $a_{y}(t_{i})$ and $a_{z}(t_{i})$ respectively represent the accelerometers output in the *x* axis, *y* axis and *z* axis at time $t_{i}$, w is the window width and $\bar{\left|a_{w}\right|}$ is the mean value of the acceleration in the window.

According to the fact that the sensor output in the ZVI is approximate to a constant, the ZVI in the human gait can be extracted by setting thresholds to Equations (10) and (11). The detail is shown in the following equation:

(12) $$\{\begin{array}{l} R_{a1}(t_{i})<\left|a(t_{i})\right|<R_{a2}(t_{i})\\ \sigma_{a}(t_{i})<R_{\sigma }(t_{i}) \end{array}$$

where $R_{a1}(t_{i})$ is the minimum threshold of the acceleration magnitude at time $t_{i}$, $R_{a2}(t_{i})$ is the maximum threshold of the acceleration magnitude at time $t_{i}$ and $R_{\sigma }(t_{i})$ is the threshold of the acceleration variance at time $t_{i}$.

When the judgment conditions in Equation (12) are fulfilled at the same time, we consider the pedestrian’s walking speed at $t_{i}$ to be approximately zero. For the traditional fixed threshold methods, $R_{a1}(t_{i})$, $R_{a2}(t_{i})$ and $R_{\sigma }(t_{i})$ are all fixed empirical values, which are only applicable to the ZVI detection at a constant walking speed rather than a changing one. In order to solve this problem and improve the robustness of the ZVI detection method, in this study, $R_{a1}(t_{i})$, $R_{a2}(t_{i})$ and $R_{\sigma }(t_{i})$ are all set as dynamic values, which are directly related to the gait frequency $f_{human\;i}$ at time $t_{i}$.

**Figure 9:**
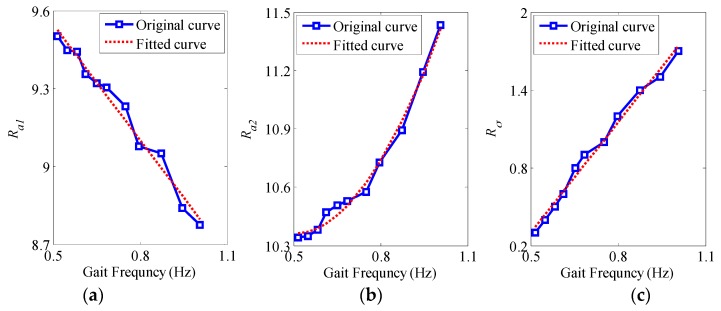
The relationships between the thresholds and the gait frequency. (**a**) The minimum threshold of the acceleration magnitude to the gait frequency; (**b**) The maximum threshold of the acceleration magnitude to the gait frequency; (**c**) The threshold of the acceleration variance to the gait frequency.

The amount of experiments on different gait frequencies have been done to obtain the relationships between the thresholds ($R_{a1}$, $R_{a2}$ and $R_{\sigma }$) and the gait frequency $f_{human}$. By collecting the gait data of the pedestrian who walks at an increasing speed and analyzing the statistical feature of the thresholds at different gait frequencies, the relationships between the thresholds and the gait frequency are shown in Figure 9.

The blue solid lines in Figure 9 are the relationship curves between the three thresholds and the gait frequency. The change tendency of the blue solid lines indicates that $R_{a1}$ decreases with the increase of the gait frequency; $R_{a2}$ and $R_{\sigma }$ increase with the increase of the gait frequency. The main cause for the changes of the three thresholds are: when the pedestrian’s walking speed increases gradually, the dynamic nature of foot movement is gradually enhanced, and the change of the signal amplitude is intensified, which leads to the corresponding changes of the ZVI detection thresholds ($R_{a1}$, $R_{a2}$ and $R_{\sigma }$). After fitting the experimental thresholds with the gait frequency in Figure 9, the relationships between the ZVI detection thresholds ($R_{a1}$, $R_{a2}$ and $R_{\sigma }$) and the gait frequency $f_{human}$ are shown as the red dotted lines. Their function expressions are:

(13) $$R_{a1}=\lambda_{1}*f_{human}+b_{1}$$

(14) $$R_{a2}=\lambda_{2}*f_{human}^{2}+\lambda_{3}*f_{human}+b_{2}$$

(15) $$R_{\sigma }=\lambda_{4}*f_{human}+b_{3}$$

where $\lambda_{1}$, $\lambda_{2}$, $\lambda_{3}$, $\lambda_{4}$, $b_{1}$, $b_{2}$ and $b_{3}$ are the fitting coefficients of the functions; meanwhile, $\lambda_{1}\;=\;-1.48$, $b_{1}\;=\;10.29$, $\lambda_{2}\;=\;4.03$, $\lambda_{3}\;=\;-4.0$, $b_{2}\;=\;11.35$, $\lambda_{4}\;=\;2.84$, $b_{3}\;=\;-1.12$. According to the function relationships between the thresholds and the gait frequency in Equations (13)–(15), the ZVI detection thresholds at different walking speeds can be obtained, and the ZVI can be detected adaptively.

## 4. Experiment Validation

In this section, we firstly give a brief description of the PNS hardware; then, the accuracy of the novel adaptive ZVI detection algorithm proposed in this paper is assessed by comparing with the traditional ZVI detection algorithm; finally, pedestrian trajectory positioning experiments at different walking speeds are carried out to evaluate the influence of the novel algorithm on positioning accuracy.

### 4.1. System Hardware Description

**Figure 10:**
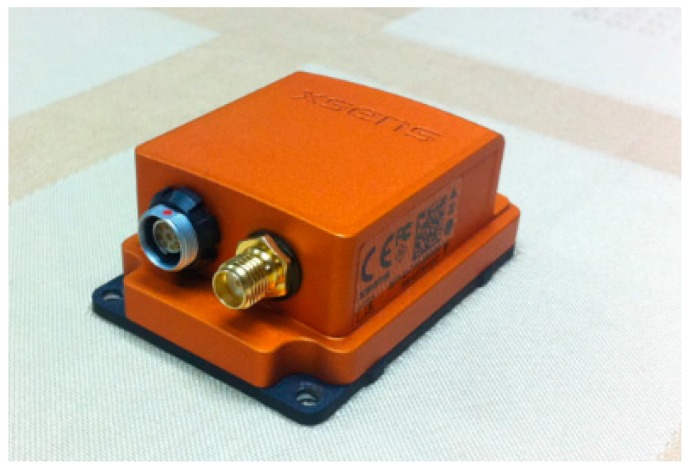
Xsens IMU for the foot-mounted PNS.

Unlike vehicles and airplanes, a pedestrian has a limited ability of carrying a load. Due to this, the IMU in the PNS has to match the characteristics of being light weight, small in size and being low power to make the pedestrian move normally, so that long time navigation can be realized. With all of the characteristics considered, the IMU MTi-G (as shown in Figure 10) produced by Xsens Technologies B.V. (Netherlands) is selected to build the testing prototype. The size of the IMU is only 57 × 42 × 24 mm, and the weight is 58 g [20]. Meanwhile, the IMU integrates three MEMS gyroscopes, three MEMS accelerometers and a triaxial magnetic sensor in a small structure. The full range of the accelerometer is ±15 g, and the gyroscope is ±1000°/s. During the experimental process, the IMU is installed on the pedestrian’s right foot, and the sampling rate is set as 100 Hz.

### 4.2. ZVI Detection Experiment

**Figure 11:**
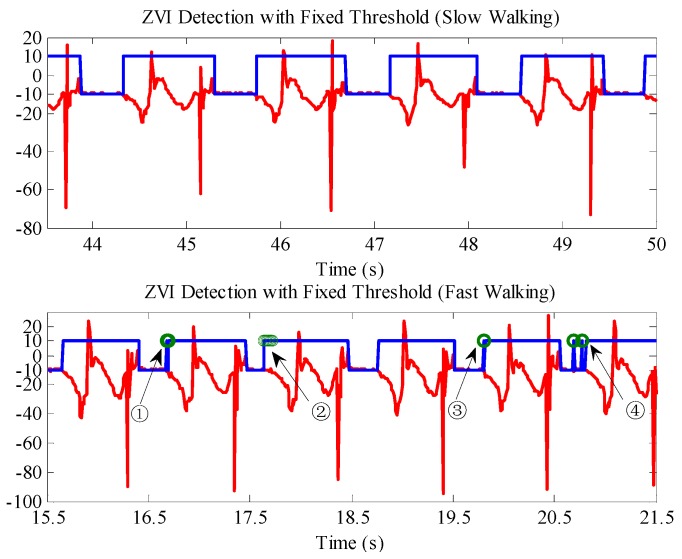
ZVI detection results with the fixed threshold method at different walking speeds. The red lines are the *z* axis accelerometer outputs. The blue lines are the ZVI detection results; −10 represents ZVI; and 10 indicates non-ZVI.

The IMU output data were collected when the experimenter walked at a slow speed (86 steps/min) and a fast speed (116 steps/min); then, with the traditional fixed threshold method, the ZVI in the pedestrian gait was detected. The upper part of Figure 11 is the ZVI detection result for slow walking. The result shows that the fixed threshold method can detect the ZVI in the human gait accurately if the thresholds are set appropriately. The bottom of Figure 11 is the ZVI detection result for fast walking, in which the selected ZVI detection thresholds are exactly the same as the fixed threshold for slow walking. The result shows that the same thresholds will lead to false and missed detections in the ZVI detection results when the walking speed changes. For example, false detections appear in the marked parts ①, ③ and ④, where the stance phase in the gait is detected as a motion state; in addition, missed detection appears in the marked part ②, where the length of ZVI in the pedestrian gait is shortened. Therefore, the fixed threshold method has a better ZVI detection effect for single walking speed, but if the pedestrian walks at different speeds, false or missed detections will appear in the ZVI detection result.

**Figure 12:**
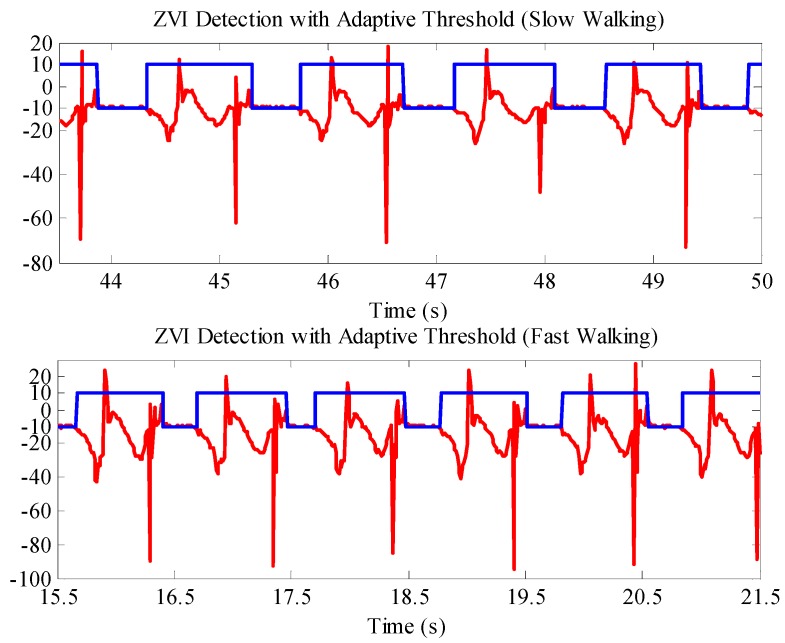
ZVI detection results with the adaptive ZVI detection algorithm at different walking speeds. The red lines are the *z* axis accelerometer outputs. The blue lines are the ZVI detection results; −10 represents ZVI; and 10 indicates non-ZVI.

Meanwhile, adopting the adaptive ZVI detection algorithm to detect the ZVI in the pedestrian gait, the results are shown in Figure 12. It can be seen that when the pedestrian walks at a slow speed, the extraction effect of ZVI by the adaptive ZVI detection algorithm is the same as adopting the fixed threshold method in Figure 11; but when the pedestrian walks at a fast speed, the novel adaptive ZVI detection algorithm can still accurately detect the ZVI without false and missed detections.

In order to further compare the ZVI detection performance between the adaptive ZVI detection algorithm and the traditional fixed threshold method, the experimenter walked 200 steps (the actual ZVI number is 100) at the speeds of 80 steps/min, 100 steps/min and 120 steps/min, respectively. Table 1 shows the results of ZVI detected by the fixed threshold method and the adaptive ZVI detection algorithm. While applying the fixed threshold method, the threshold of 100 steps/min (Th100) is selected as the detection threshold for the three walking speeds.

The data in Table 1 indicate that Th100 has the best ZVI detection result for a 100-steps/min walking speed, and the detected ZVI number is 100, which is the same as the actual ZVI number. For the other two walking speeds, 80 steps/min and 120 steps/min, the ZVI detected by the fixed threshold method includes two false detections and six missed detections, respectively. However, the ZVI number detected by the adaptive ZVI detection algorithm is 100 for the three different walking speeds, the same as the actual ZVI number. Its root cause lies in the fact that the proposed method can adaptively provide optimal thresholds for ZVI detection with the gait frequency changing, which avoids the limited application of the fixed threshold only for a single walking speed and effectively reduces the rate of false and missed detections.

Therefore, the design and implementation of the adaptive ZVI detection algorithm enhances the accuracy of ZVI detection at different walking speeds, which further improves the positioning effect of PNS and makes it have better robustness and stronger practical application.

### 4.3. Pedestrian Trajectory Positioning Experiment

Furthermore, pedestrian trajectory positioning experiments at different walking speeds were carried out to assess the influence of the novel adaptive ZVI detection algorithm on positioning accuracy.

#### Rectangular Route Experiment

**Figure 13:**
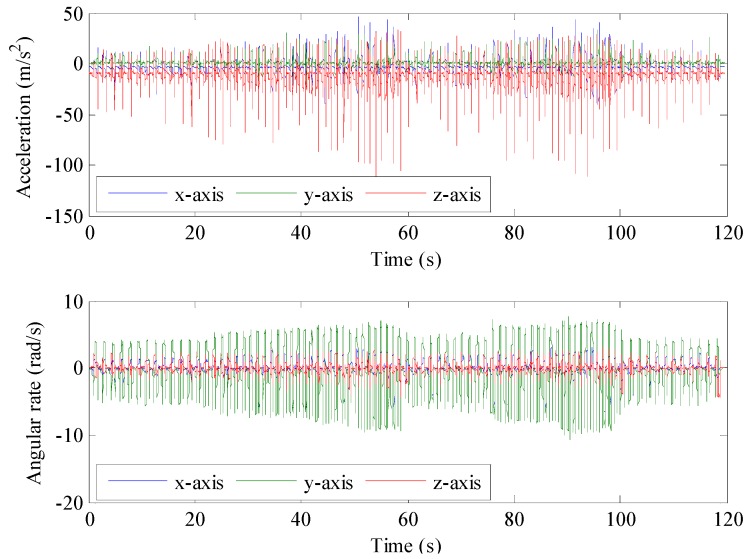
Output of the IMU during walking.

The pedestrian trajectory positioning experiment was carried out on a 49 m-wide and 13.9 m-long rectangular route on campus. Facing north, the experimenter started and walked a loop along the route at “slow-normal-fast-slow-normal-fast-slow” speed alternately and finally returned to the start point. The total walking distance was 125.8 m, and the total walking time was 119.6 s. The raw data output by the accelerometers and gyroscopes during the walking process are shown in Figure 13, indicating that the accelerometer in the *z* axis has a better effect on sensing the foot movement and that the amplitude of the acceleration changes greatly; the gyroscope in the *y* axis has an obvious perception of foot rotation, and the amplitude of the angular rate changes greatly. In addition, it can also be found from Figure 13 that the amplitude and gait frequency of the sensors’ output change with the walking speed. When the walking speed increases, the amplitude and gait frequencies increase, and vice versa.

**Figure 14:**
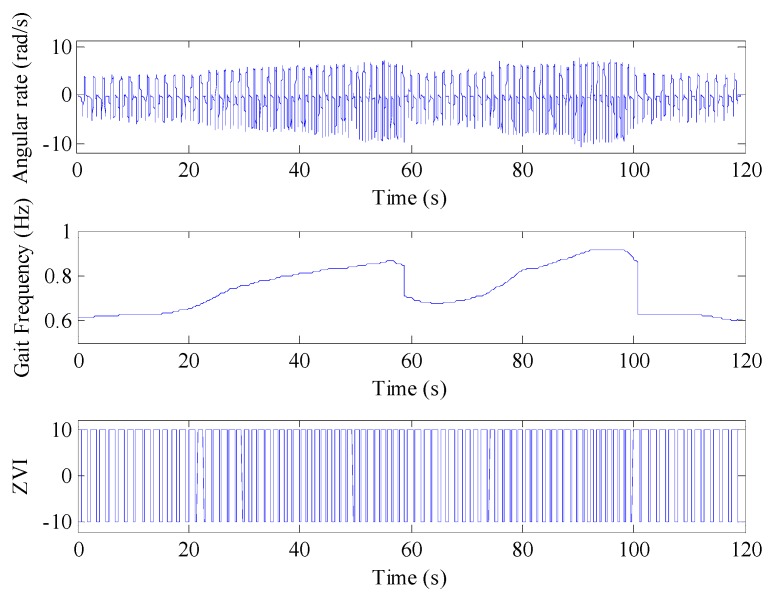
Gait frequency and ZVI detection results. In the bottom of the figure, −10 represents ZVI and 10 indicates non-ZVI.

By adopting the adaptive ZVI detection algorithm to analyze the IMU output during the walking process, the detection results of the gait frequency and ZVI are provided in Figure 14. The upper part of Figure 14 is the *y* axis gyroscope output, showing that the amplitude and the cycle of the angular rate change with the walking speed, which matches the “slow-normal-fast-slow-normal-fast-slow” speed changing characteristic. The middle part of Figure 14 shows the real-time gait frequency extracted by the adaptive ZVI detection algorithm. It can be seen that the time-frequency curve clearly and accurately reflects the gait frequency change during the walking process, which provides the real-time gait frequency for selecting dynamic thresholds in the process of adaptive ZVI detection. The bottom of Figure 14 is the ZVI detected by the adaptive ZVI detection algorithm, and the result shows that there is no false and missed detection during the walking process.

**Figure 15:**
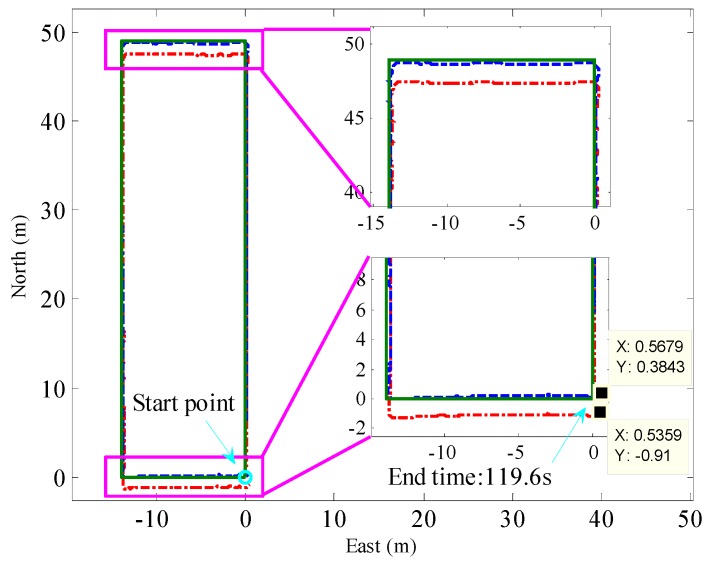
Results of walking along a rectangle trajectory. The red dashed-dotted line and the blue dashed line represent the trajectory calculated by the ZUPT-aided pedestrian navigation algorithm with the traditional fixed threshold method and the adaptive ZVI detection algorithm, respectively. The green solid line is the reference trajectory.

Then, using the ZUPT-aided pedestrian navigation algorithm to calculate the pedestrian trajectory. The results are shown in Figure 15. The red dashed-dotted line and the blue dashed line represent the trajectories calculated by the ZUPT-aided pedestrian navigation algorithm with the traditional fixed threshold method and the adaptive ZVI detection algorithm, respectively. The green solid line is the reference trajectory. The local enlarged drawings of Figure 15 show that after obtaining the ZVI by the fixed threshold method, the calculated trajectory deviates from the reference trajectory, and the position error is more than 1 m in the east-west direction. The main cause of the position error is that there exist false and missed detections of ZVI with the fixed threshold method when the experimenter walks at different speeds. However, after obtaining the ZVI by the adaptive ZVI detection algorithm, the calculated trajectory fits with the reference trajectory well. The position error at the end point is 0.69 m, accounting for 0.55% of the total walking distance. Thus, the ZVI detected by the adaptive ZVI detection algorithm for pedestrian trajectory calculation can achieve higher positioning accuracy.

**Figure 16:**
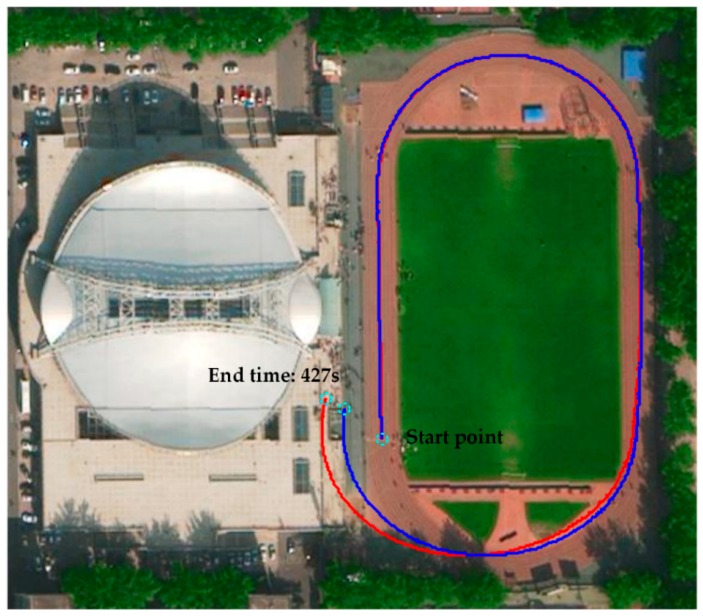
Results of walking around the playground in a circle. The red line represents the pedestrian trajectory calculated by the ZUPT-aided pedestrian navigation algorithm with the fixed threshold method; the blue line is the pedestrian trajectory calculated by the ZUPT-aided pedestrian navigation algorithm with the adaptive ZVI detection algorithm.

We also conducted the pedestrian trajectory positioning experiment on a longer path, as shown in Figure 16. The experimenter walked a loop on the campus runway at “normal-slow-fast-slow” walking speed. The total walking distance was about 430 m, and the total walking time was 427 s. The red and blue curves are the pedestrian trajectories calculated by the ZUPT-aided pedestrian navigation algorithm with the fixed threshold method and the adaptive ZVI detection algorithm, respectively. From Figure 16, we can learn that the pedestrian position error by adopting the fixed threshold method (5.1% of the travelled distance) is larger than the one with the adaptive threshold algorithm (3.5% of the travelled distance). The reason is that when the experimenter walks at different speeds, false or missed detections appear when applying the fixed threshold method, which further leads to false or missed corrections in the ZUPT process. However, the adaptive ZVI detection algorithm can adjust the thresholds in real time according to the gait frequency, which ensures the proper execution of ZUPT and can correct the navigation error adequately. In addition, the trajectories calculated by the two methods share the common characteristics that the heading drifts gradually with the increase of the walking distance, the reason for which is that ZUPT fails to correct the heading error [21,22], leading to the accumulation of heading error calculated by SINS. Heading drift, another important problem in the PNS, is an issue that will be discussed in a future study.

## 5. Conclusions

This paper designs a novel adaptive ZVI detection algorithm based on SPWVD-RMFI for the PNS with self-contained sensors. The novel algorithm can extract the pedestrian’s gait frequency during walking in real time. With the establishment of the function relationships between ZVI detection thresholds and gait frequency, the problem of setting the thresholds adaptively for ZVI detection at different walking speeds is solved, which realizes the accurate detection of ZVI. Compared with the traditional fixed threshold method, the novel algorithm has better robustness and higher detection precision of ZVI. At the same time, the results of ZVI detection contrast experiments and pedestrian trajectory positioning experiments at different walking speeds show that the novel algorithm can effectively suppress the false and missed detections existing in the traditional fixed threshold method. Meanwhile, the ZVI detected by the adaptive ZVI detection algorithm for pedestrian trajectory calculation has better performance. In addition, the SPWVD-RMFI algorithm presented in this paper can also be applied to the motion frequency detection of human health monitoring and the step length estimation of PNS based on the pedestrian dead reckoning algorithm.

In a future study, we will pay close attention to the problem of heading correction for PNS. By adopting the adaptive ZVI detection algorithm proposed in this paper for ZUPT and combing the heading correction algorithm, the positioning accuracy of the PNS can be further improved.

## Figures and Tables

**Table 1:** ZVI detection results at different walking speeds.

Walking Speed (Steps/min)	Actual ZVI Number	Detected ZVI Number	
		Fixed Threshold Method	Adaptive ZVI Detection Algorithm
80	100	102	100
100	100	100	100
120	100	94	100
